# Short-Range Correlated Magnetic Core-Shell CrO_2_/Cr_2_O_3_ Nanorods: Experimental Observations and Theoretical Considerations

**DOI:** 10.3390/nano8050312

**Published:** 02018-05-09

**Authors:** Ashish C. Gandhi, Tai-Yue Li, Ting Shan Chan, Sheng Yun Wu

**Affiliations:** 1 Department of Physics, National Dong Hwa University, Hualien 97401, Taiwan; acg.gandhi@gmail.com (A.C.G.); tim312508@gmail.com (T.-Y.L.); 2 National Synchrotron Radiation Research Center, Hsinchu 300, Taiwan; chan.ts@nsrrc.org.tw

**Keywords:** nanocrystalline materials, CrO_2_/Cr_2_O_3_, core-shell nanorods, oxide materials

## Abstract

With the evolution of synthesis and the critical characterization of core-shell nanostructures, short-range magnetic correlation is of prime interest in employing their properties to develop novel devices and widespread applications. In this regard, a novel approach of the magnetic core-shell saturated magnetization (CSSM) cylinder model solely based on the contribution of saturated magnetization in one-dimensional CrO_2_/Cr_2_O_3_ core-shell nanorods (NRs) has been developed and applied for the determination of core-diameter and shell-thickness. The nanosized effect leads to a short-range magnetic correlation of ferromagnetic core-CrO_2_ extracted from CSSM, which can be explained using finite size scaling method. The outcome of this study is important in terms of utilizing magnetic properties for the critical characterization of core-shell nanomagnetic materials.

## 1. Introduction

A usual approach in nanoscience generally involves two steps: first, the fabrication and characterization of nanomaterials, and second, employing their properties for the development of novel devices and widespread applications. In recent years, the fabrication and critical characterization of core-shell nanostructures has attracted enormous attention because of their widespread applications [1,2,3,4,5]. The enormous progress made in the field of high-density recording and nanotechnology make the study of exchange bias (EB) ferromagnetic (FM)/Antiferromagnetic (AF) core/shell system important. Among different EB systems, exchange coupled CrO_2_/Cr_2_O_3_ are the least explored nanostructures due to difficulty in the critical characterization of constitute phases [6,7,8]. CrO_2_ is a half-metallic ferromagnetic (FM) [9] material with a Curie temperature (*T_C_*) of ~394 K, well above room temperature, which was used widely as an information storage material for magnetic recording tapes [10,11]. Recently, the half-metallic properties [12] of CrO_2_ such as spin-dependent magnet-transport [13,14], double exchange coupling interactions due to the self-doping effect [15], the large magnetoresistance effect [16,17], and anomalous transport properties [18], etc., have attracted enormous interest. On the other hand, Cr_2_O_3_ is a low-anisotropy easy axis antiferromagnetic (AF) material with a Neel temperature (*T_N_*) of ~307 K [19,20]. It exhibits piezomagnetic and magnetoelectric effects, and can be used in a device involving EB coupling, which makes it a promising material for the future spintronic-based applications [21,22,23]. The core-shell CrO_2_/Cr_2_O_3_ nanostructures can easily be formed even at room temperature simply by the surface reduction of CrO_2_ to Cr_2_O_3_ when exposed to air for a prolonged period of time [24]. Such an insulating Cr_2_O_3_ surface layer was treated as a tunneling barrier between CrO_2_ particles by EB coupling [25]. The interface-driven magnetoelectric effect has also been reported from granular CrO_2_ due to the formation of AF-Cr_2_O_3_ surface layer [26,27]. The core diameter $d_{core}$ of CrO_2_ and shell-thickness $t_{shell}$ of Cr_2_O_3_ can be further tuned simply by a thermal treatment in an ambient atmosphere at an elevated temperature. With the increase of reduction temperature, the long-range AF spin correlation of the Cr_2_O_3_ phase strengthens at the expense of the core CrO_2_ phase, resulting in a short-range FM spin correlation. Therefore, due to the reduction of CrO_2_ to Cr_2_O_3_, the magnetic configuration of the system is expected to experience the coexistence and competition of AF and FM interactions. However, the most difficult part is its critical characterization, particularly estimating the values of $d_{core}$ and $t_{shell}$ parameters. 

In this work, the effect of thermal treatment resulting in the surface reduction of CrO_2_ nanorods (NRs) to Cr_2_O_3_ phase is studied via the Rietveld refined X-ray diffraction (XRD) spectra and magnetic measurements. We have demonstrated utilization of magnetic properties of FM-CrO_2_ as a tool to estimate the values of $d_{core}$ and $t_{shell}$ of CrO_2_/Cr_2_O_3_ core-shell NRs. The observed finite size scaling behavior from core diameter $d_{core}$ the dependency of *T_C_*, confirmed the validity of used magnetic property-based theoretical expression. 

## 2. Synthesis of CrO_2_/Cr_2_O_3_ Nanostructures

In the synthesis of CrO_2_-core with Cr_2_O_3_-shell NRs, we have carried out the reduction of pristine CrO_2_ NRs simply by thermal treatment at 450, 500, 550, and 600 °C for a duration of 1 h in an ambient atmosphere. The pristine CrO_2_ NRs were obtained commercially from Sigma-Aldrich (St. Louis, MO, USA) under the name of Magnetrieve. The reduction is carried out in a tube furnace with a heating rate of 5 K/min and allowed to cool down naturally. Note that thermal treatment at and above 650 °C resulted in a complete reduction of CrO_2_ into pure AF Cr_2_O_3_ phase [28]. Structural properties were studied using synchrotron radiation XRD (SRXRD) (λ = 0.7749 Å) at National Synchrotron Radiation Research Center (NSRRC) beamline BL01C2 in Taiwan. Further analysis of the structural properties was carried out by selective area diffraction pattern (SAED) using JEOL 2010 TEM (Peabody, MA, USA) working at 200 kV. The analysis of SAED pattern was done by CaRIne Crystallography 3.1 software. Field-emission scanning electron microscopy (FE-SEM, JEOL JSM-6500F, Peabody, MA, USA) was utilized for the estimation of diameter distribution and morphological analysis. The mean diameter distribution was estimated from relative SEM images using Nano Measurer version 1.2.5. The temperature dependences of zero-field-cooled (ZFC) and field-cooled (FC) magnetization, thermoremanent magnetization (TRM) decay, and isothermal field dependences of magnetization measurements were carried out using a quantum design MPMS-SQUID-VSM magnetometer (San Diego, CA, USA).

## 3. Results and Discussion

### 3.1. X-ray Diffraction Analysis 

**Figure 1:**
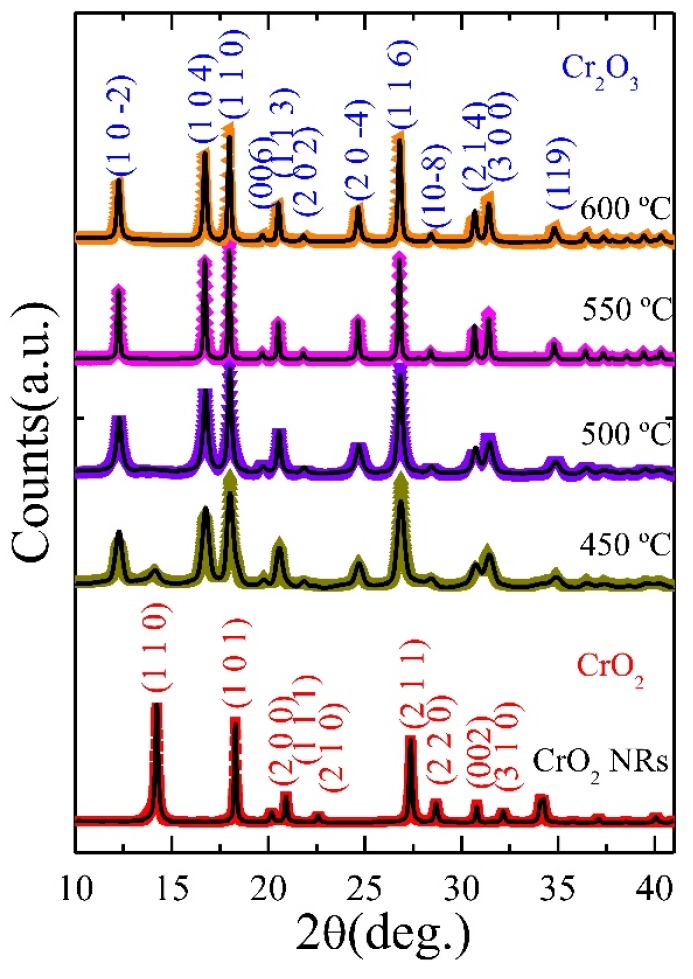
Synchrotron radiation XRD spectra of pristine CrO_2_ and 450 °C to 600 °C NRs (bottom to top), in which solid curve represents the Rietveld fit to the SRXRD spectrum.

Figure 1 displays the SRXRD spectra for the pristine CrO_2_ NRs and 450, 500, 550, and 600 °C samples (spectra are shifted vertically for clear visibility). The diffraction spectrum of the pristine NRs is assigned to pure CrO_2_ phase indexed with space group P4_2_/mnm [25]. Along with CrO_2_ phase, SRXRD spectra of 450 °C sample display additional peaks assigned to Cr_2_O_3_ phase indexed with space group *R*3-*c* [28]. 500, 550, and 600 °C samples do not show any noticeable diffraction peaks for CrO_2_ phase, as can be clearly visualized from magnified spectrum shown in the supporting Figure S1a. For further detailed structural analysis, Rietveld [29] refinement of SRXRD spectra was carried out using GSAS [30] software package. The solid black line in Figure 1 is the fitted curve, and fitting parameters are tabulated in Supporting Table S1. From Rietveld refinement, we confirm that (i) only CrO_2_ phase (*a* = *b* = 4.4215 Å, *c* = 2.9177 Å) existed in the pristine sample; (ii) both CrO_2_ (*a* = *b* = 4.4715 Å, *c* = 2.9206 Å; weight fraction 0.33%) and Cr_2_O_3_ (*a* = *b* = 4.9595 Å, *c* = 13.5684 Å; weight fraction 99.67%) phases existed in 450 °C sample; and (iii) only Cr_2_O_3_ phase existed in 500 to 600 °C samples. The variation in the lattice constant (*a* = *b*, *c*) of Cr_2_O_3_ phase with respect to the annealing temperature (*T_A_*) is depicted in supporting Figure S1b. As compared to bulk Cr_2_O_3_ (*a* = *b* = 4.959 Å, *c* = 13.589 Å), sample reduced at 450 °C shows a noticeable lattice contraction along *c*-axis, whereas lattice constants along *a*-, *b*-axis have a value close to bulk. The observed contraction along *c*-axis could be attributed to finite size effect or defects [28].

### 3.2. Morphological and Structural Analysis

**Figure 2:**
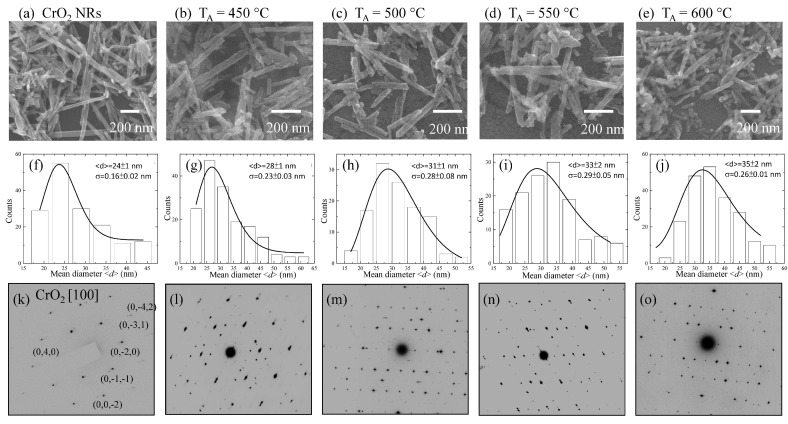
(**a**–**e**) SEM images; (**f**–**j**) histogram of diameter distribution and (**k**–**o**) SAED patterns of the CrO_2_/Cr_2_O_3_ core-shell NRs. The line in (**f**–**j**) represents fit to the histogram using log-normal distribution function.

The SEM images in Figure 2a–e show the nanorod-like morphology of pristine and 450, 500, 550, and 600 °C samples, respectively. The effect of reduction resulted in the increase of NRs diameter, but with relatively smaller length than that of pristine NRs. The mean diameter <*d*> of the asymmetrically distributed NRs can be obtained by fitting log-normal distribution function:

$$f\left(d\right)=\frac{1}{\sqrt{2\pi }d\sigma }\exp\left[-\frac{{\left(\ln d-\ln<d>\right)}^{2}}{2\sigma^{2}}\right]$$

in which *σ* is a standard deviation of the fitted function to the histogram of NRs diameter obtained from SEM images (Figure 2f–j). The fitted values of (<*d*>, *σ*) of CrO_2_ NRs and 450, 500, 550, and 600 °C samples are (24 ± 1 nm, 0.16 ± 0.02 nm), (28 ± 1 nm, 0.23 ± 0.03 nm), (31 ± 1 nm, 0.28 ± 0.08 nm), (33 ± 2 nm, 0.29 ± 0.05 nm), and (35 ± 2 nm, 0.26 ± 0.01 nm), respectively. As an effect of the increase of *T_A_*, the mean diameter of CrO_2_/Cr_2_O_3_ NRs increases and the diameter distribution widens. These changes could also be accompanied by an increase of reduction rate of CrO_2_ to Cr_2_O_3_ with the increase of *T_A_* [31]. Note that thermal reduction at 650 °C resulted in the formation of pure Cr_2_O_3_ NRs with <*d*> = 35 ± 1 nm [28].

For further structural characterization, the SAED pattern of pristine NRs, reduced samples, and Cr_2_O_3_ NRs [28] was examined. The SAED patterns in Figure 2k can be ascribed to the tetragonal-CrO_2_ oriented along the [100] zone axis. Interestingly, along with 450 °C, the SAED pattern of 500, 550, and 600 °C samples displayed the existence of mix crystalline phases, as shown in Figure 2l–o. The observed mix phases in CrO_2_-reduced samples were ascribed to the tetragonal-CrO_2_ and hexagonal-Cr_2_O_3_ phases oriented along the [100] and [−101] zone axis, respectively. However, observed mixed phase SAED pattern of 500, 550, and 600 °C samples contradict with the SRXRD spectra in which only Cr_2_O_3_ phase was noted, since SAED is a local technique and hence gives structural information only from a limited area. On the contrary, SRXRD, which is a global technique, gives average structural information. In 500 to 600 °C reduced samples, the weight percent of CrO_2_ is <0.33%, which is far below the SRXRD detection limit of ~1%. Similar discrepancy was also reported in our previous work on 14 nm Ni/NiO nanoparticles in which tiny amount of Ni phase (<0.7%) was detected through SAED and static/dynamic magnetic measurements but was hard to detected using SRXRD [32]. Since magnetic moment of FM-CrO_2_ is about 10,000 times that of AF-Cr_2_O_3,_ in order to gain a detailed understanding of thermal reduction effects on CrO_2_ NRs, we have carried out both field- and temperature-dependent magnetization measurements.

### 3.3. Determination of Core-Diameter and Shell-Thickness from the Saturated Magnetization

**Figure 3:**
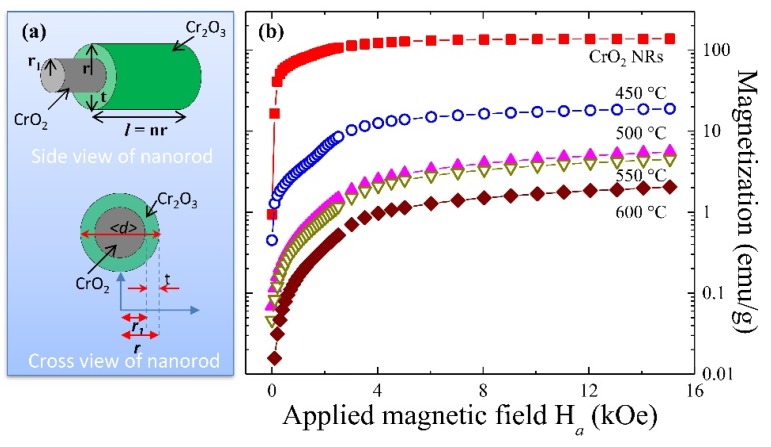
(**a**) A schematic illustration of side- and cross-view of CrO_2_/Cr_2_O_3_ core-shell nanorod; (**b**) magnetization versus applied magnetic field of CrO_2_ and 450 to 600 °C NRs measured at 2 K from 0 to 15 kOe.

In the core-shell magnetic nanomaterials, usually, a large variety of possible magnetic behaviors can be encountered that vary with the type of material, the magnetic system (FM, ferrimagnetic, AF, superparamagnetic, etc.), inter- and intra-particle interactions, interface coupling, size, and morphologies. In such complex systems, it very difficult to distinguish the intrinsic physical properties with respect to the size that is often obtained only from few nanowires or nanoparticles using TEM or SEM images. Therefore, from the macroscopic point of view, necessity of a characteristic magnetic ‘fingerprint’ arises such that different magnetic core-shell systems can be classified and distinguished to determine the core-diameter and shell-thickness. Saturated magnetization is a good candidate for a fingerprint for the FM-AF core-shell nanosized system. In general, according to Néel, the thermal fluctuations will affect the magnetization of a single domain ferromagnetic particle, resulting in its decay towards the thermal equilibrium. However, he has neglected the contribution from magnetic anisotropy. Later, Néel-Brown introduces a thermally activated magnetization reversal model to describe a single domain magnetic particle with two equivalent ground states of opposite magnetization separated by an energy barrier, which is due to the shape and crystalline anisotropy. Within the framework, we establish a core-shell saturated magnetization (CSSM) cylinder model to describe the moment contribution in saturated magnetization at the lowest temperature. A schematic of the side view and cross view of thermally reduced core-shell nanorod is shown in Figure 3a, such that atoms of Cr_2_O_3_-shell and CrO_2_-core give rise to a net surface macro-moment m_2_ and a net inner macro-moment m_1_. Quantitative analysis for the CSSM was applied to the saturated magnetization $M_{S}$ at various annealing temperatures *T_A_*, as shown in Figure 3b. In the theoretical modeling, we assume that contribution from the magnetic moments of Cr_2_O_3_ phase to the saturation magnetization ($M_{S}$) in CrO_2_/Cr_2_O_3_ core-shell NRs is very weak, such that it can be neglected, and therefore only FM-CrO_2_ phase contributes to the $M_{S}$. We propose that the saturated magnetization at the lowest temperature, which was computed from the saturated moment divided by the mass of CrO_2_ content from elemental analysis, can be described as

(1) $$\frac{M_{S}}{M_{S}^{CrO_{2}}}=\left[\frac{N_{CrO_{2}}}{N_{CrO_{2}}+N_{Cr_{2}O_{3}}}\right]$$

in which $M_{S}^{CrO_{2}}$ is the saturation magnetization of CrO_2_ NRs. $N_{CrO_{2}}$ and $N_{Cr_{2}O_{3}}$ are the average number of CrO_2_ and Cr_2_O_3_ atoms per gm at the core and in the shell, which can be calculated using bulk densities (*δ*) of CrO_2_ and Cr_2_O_3_ (4.89 g/cm^3^, 5.22 g/cm^3^) as

(2) $$N_{CrO_{2}}=\delta_{CrO_{2}}V_{CrO_{2}}N_{A}$$

(3) $$N_{Cr_{2}O_{3}}=\delta_{Cr_{2}O_{3}}V_{Cr_{2}O_{3}}N_{A}=c\delta_{CrO_{2}}V_{Cr_{2}O_{3}}N_{A}$$

in which $c=\frac{\delta_{Cr_{2}O_{3}}}{\delta_{CrO_{2}}}$; *N_A_* is Avogadro’s number. Combining Equations (1)–(3) gives

(4) $$\frac{V_{CrO_{2}}}{V_{CrO_{2}}+cV_{Cr_{2}O_{3}}}=\frac{M_{S}}{M_{S}^{CrO_{2}}}$$

considering that a CrO_2_/Cr_2_O_3_ core-shell NRs has a concentric cylinder, the total radius (*r*) will be given by the sum of core radius (*r*_1_) and shell thickness (*t*), as shown in Figure 3a. The volume of a cylinder is given by $V=\pi r^{2}l$, in which l is a length of the nanorod. Considering *n* as any positive number, the length of a cylinder can be expressed as l=nr. The volume of core and shell can be written separately as $V_{CrO_{2}}=\pi r_{1}^{2}\left(nr-2t\right)$ and $V_{Cr_{2}O_{3}}=\pi nr^{3}-V_{CrO_{2}}=\pi nr^{3}-\pi r_{1}^{2}\left(nr-2t\right)$, respectively, and, combined with Equation (4), we can obtain a relation between the thickness and saturated magnetization as

(5) $$t=r\left[1-\frac{1}{\sqrt{\frac{\left(nr-2t\right)}{nr}\left[\left(\frac{M_{S}^{CrO_{2}}}{cM_{S}}-\frac{1}{c}\right)+1\right]}}\right]$$

The nanorod length is much larger than the shell thickness; we can take (nr−2t)≈nr, which resulted in the simplified CSSM model as

(6) $$t=r\left[1-\frac{1}{\sqrt{\left(\frac{M_{S}^{CrO_{2}}}{cM_{S}}-\frac{1}{c}\right)+1}}\right]$$

**Figure 4:**
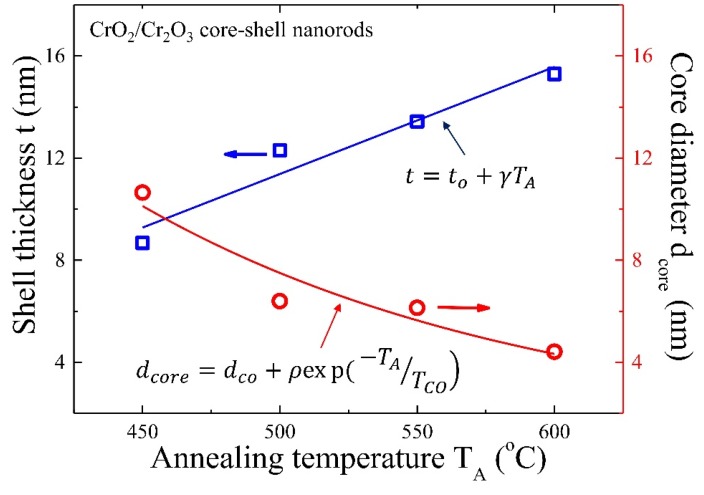
A plot of the *T_A_* dependence of the obtained shell thickness *t* and the core diameter $d_{core}$. The solid curves represent linear and exponential fits to the experimental data.

Above expression gives the useful relation between the Cr_2_O_3_ shell thickness *t* and the total radius r=dcore2+t of CrO_2_/Cr_2_O_3_ core-shell NRs. The above simplified CSSM model was used to calculate Cr_2_O_3_ shell thickness and CrO_2_ core diameter as tabulated in Supporting Table S2. As shown in Figure 4, the core diameter $d_{core}$ decreases with the increase of annealing temperature *T_A_*, which can be described very well by fitting an exponential decay function dcore=dco+ρexp(−TATCO), in which $d_{co}$ = 1.13 ± 0.03 nm, *ρ* = 200 nm, and $T_{CO}$ = 145 ± 12 °C represent the initial constants and the fitted parameters, respectively. The observed linear increasing behavior of Cr_2_O_3_ shell thickness can be described using growth function $t=t_{o}+\gamma T_{A}$, in which $t_{o}$ = −9.6 ± 3.8 nm and *γ* = 0.042 ± 0.007 nm/°C. The above finding indicates that the surface reduction of CrO_2_ to Cr_2_O_3_ begins around *T_A_* ~229 °C, and a self-terminated layer of ~17.5 nm was obtained at *T_A_* ~650 °C [28]. It is noteworthy that the shell thickness can also be estimated from TEM results, but with less accuracy, because CrO_2_ NRs on TEM substrate film are more susceptible to oxidation than samples for magnetometer measurement that were stored in a capsule. 

### 3.4. Temperature Dependence of Thermoremanent Magnetization (TRM)

**Figure 5:**
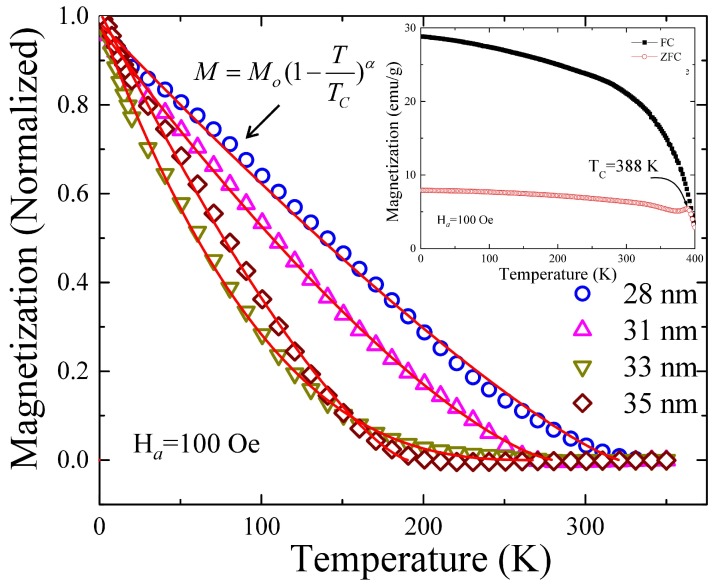
Temperature dependency of thermoremanent magnetization of 450 to 600 °C NRs. The solid curve represents a fit using a model given in the text. Inset of the figure shows the ZFC-FC curve of pristine CrO_2_ NRs.

In general, in a superparamagnetic (SPM) nanoscaled system, thermoremanence is related to the distribution of energy barriers. At a given measuring temperature and after removing the applied field, only the particles that are in the blocked regime will contribute to the remanent magnetization. To estimate the effect of thermal treatment on *T_C_* of CrO_2_ phase, TRM decay measurements of 450 to 600 °C samples were undertaken. Initially, sample was cooled down from 350 to 1.8 K under an applied magnetic field of *H_a_* = 100 Oe. Once temperature stabilized, the magnetic field was switched off using oscillating mode, and subsequently magnetization decay with respect to temperature *T* was recorded while warming the sample in a zero field. Figure 5 shows the normalized plot of TRM curve, indicating the presence of irreversible contributions in the low-temperature region. A monotonically decreasing behavior reflecting the expected thermally induced decay of magnetization was observed and vanished above a characteristic temperature. The value of *T_C_* from CrO_2_/Cr_2_O_3_ core-shell NRs was obtained by fitting TRM curve using M(T)=Mo(1−TTC)α, in which α is a power law factor. The solid line in Figure 5 represents the fitted curve using above expression, and fitting parameters are tabulated in Supporting Table S2. The fitted value of *T_C_* matches with ferromagnetic ordering temperature, which marks the ordering temperature of the FM cores, i.e., 321 ± 5, 281 ± 5, 271 ± 15, and 191 ± 14 K for <*d*> = 28, 31, 33, and 35 nm NRs, respectively. The amount of reduction of CrO_2_ to Cr_2_O_3_ increases with the increase of *T_A_* up to 600 °C, above which a pure Cr_2_O_3_ phase was obtained. Therefore, observed decreases of *T_C_* with an increase se of *T_A_* could be resulted from the finite size effect, which will be discussed further in the text. The value of *T_C_* ~388 K of pristine CrO_2_ NRs is defined from a point at which ZFC-FC curve overlaps and above which it retains superparamagnetic behavior such as magnetization decay with the increase of *T* as shown in the inset of Figure 5. Note that obtained value of *T_C_* from pristine CrO_2_ NRs is very close to the report value of 394 K. 

### 3.5. Finite Size Scaling Method

**Figure 6:**
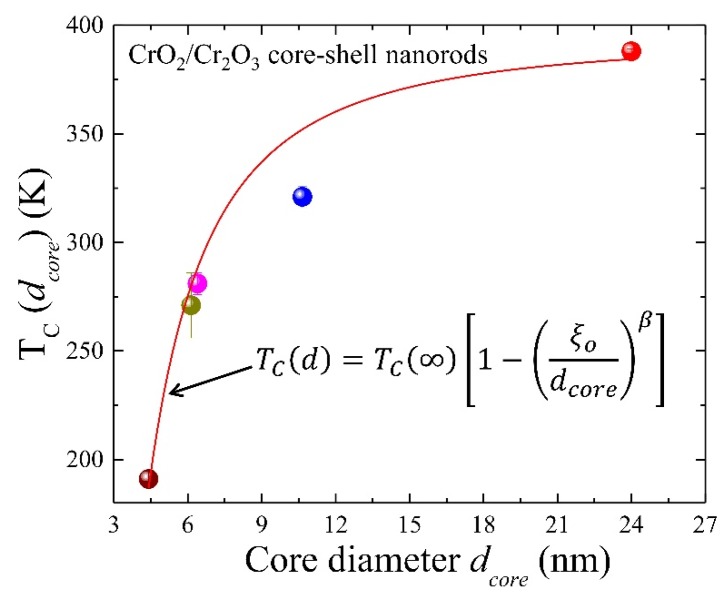
A plot of core diameter $d_{core}$ dependence of the *T_C_*, in which solid curve represents a fit using finite size scaling model discussed in the text.

The values of *T_C_* decrease from 388 to 191 K with the decrease of CrO_2_ diameter $d_{core}$ from 24 to 4.42 nm as shown in Figure 6, signaling a strong size effect. In a nanoscaled FM-core system, the finite size scaling method can be used to extract the values for the critical exponents by observing how measured quantities vary as the size *d* of the system studied changes. However, the technique we used for those calculations required us to perform simulations exactly at the critical temperature of the model, which in turn requires us to know *T_C_*. The diameter dependence of *T_C_* can be explained using finite size scaling formula [33] $T_{C}\left(d\right)=T_{C}\left(\infty \right)\left[1-{\left(\frac{\xi_{o}}{d_{core}}\right)}^{\beta }\right]$, in which $T_{C}\left(\infty \right)$ = 394 K for bulk CrO_2_, $\xi_{o}$ is the characteristic microscope dimension of the system at zero temperature, and the exponent is related to the correlation length by *β* = 1/*λ*. Fitting *T_C_* vs. $d_{core}$ of core-shell NRs yields $\xi_{o}$ = 3.1 ± 0.2 nm, which is roughly ~0.70 and ~1.06 times the lattice constant *a* = *b* and *c*. The value of the characteristic microscopic length scale indicates that growth of CrO_2_ NRs is along the *c*-axis. The obtained value *λ* = 0.56 (*β* = 1.8 ± 0.2) is close to the value predicted using isotropic 3D Heisenberg model (0.65 ≤ λ ≤ 0.733) [34]. Above finding shows that the finite size scaling formula can efficiently describe the shift in *T_C_*, giving validation of used magnetic property-based theoretical expression for the estimation of core and shell parameters.

## 4. Conclusions

The series of CrO_2_/Cr_2_O_3_ core-shell NRs with mean diameters varying from 28 to 35 nm were synthesized simply by thermal reduction technique. A new approach by means of DC magnetometer and a core-shell saturated magnetization (CSSM) cylinder model has been proposed for the estimation of core-CrO_2_ diameter and shell-Cr_2_O_3_ thickness, which is otherwise impossible to calculate using any conventional means. We observed that with the increase of reduction temperature from 450 to 600 °C, core-CrO_2_ diameter reduced from ~11 to 4 nm and shell-Cr_2_O_3_ thickness increased from ~9 to 15 nm, respectively. Finite size effect of FM-CrO_2_ leads to reduced Curie temperature *T_C_* of NRs, which can be explained further using the finite size scaling model, giving a short-range magnetic correlation length of = $\xi_{o}$ 3.1 ± 0.2 nm. This work is believed to be of importance, especially for researchers working on the magnetic core-shell nanostructures and their application in functional devices.

## Supplementary Materials

The following are available online at http://www.mdpi.com/2079-4991/8/5/312/s1.
