# Supplementary material for: Short-Range Correlated Magnetic Core-Shell CrO2/Cr2O3 Nanorods: Experimental Observations and Theoretical Considerations
Source: Nanomaterials (Basel). 2018 May 9;8(5):312. doi: 10.3390/nano8050312 (PMC5977326; doi:10.3390/nano8050312)
Supplement: Supplementary file 1 [file nanomaterials-08-00312-s001.pdf]

Supporting information

Short Range Correlated Magnetic Core-Shell CrO_2/Cr_2O_3 Nanorods: Experimental observations and theoretical considerations

Ashish C. Gandhi,[1] Tai-Yue Li,[1] Ting Shan Chan,[2] and Sheng Yun Wu[1,*]

[1] Department of Physics, National Dong Hwa University, Hualien 97401, Taiwan

[2] National Synchrotron Radiation Research Center, Hsinchu, Taiwan

Figure S1. (a) Magnified XRD spectra of CrO_2, 450 °C and 500 °C NRs. **(b)** Plot of T_A dependence of lattice constants of Cr_2O_3 phase, where dashed-dot and dashed lines represent the lattice constant $a = b$ and c of bulk Cr_2O_3, respectively.

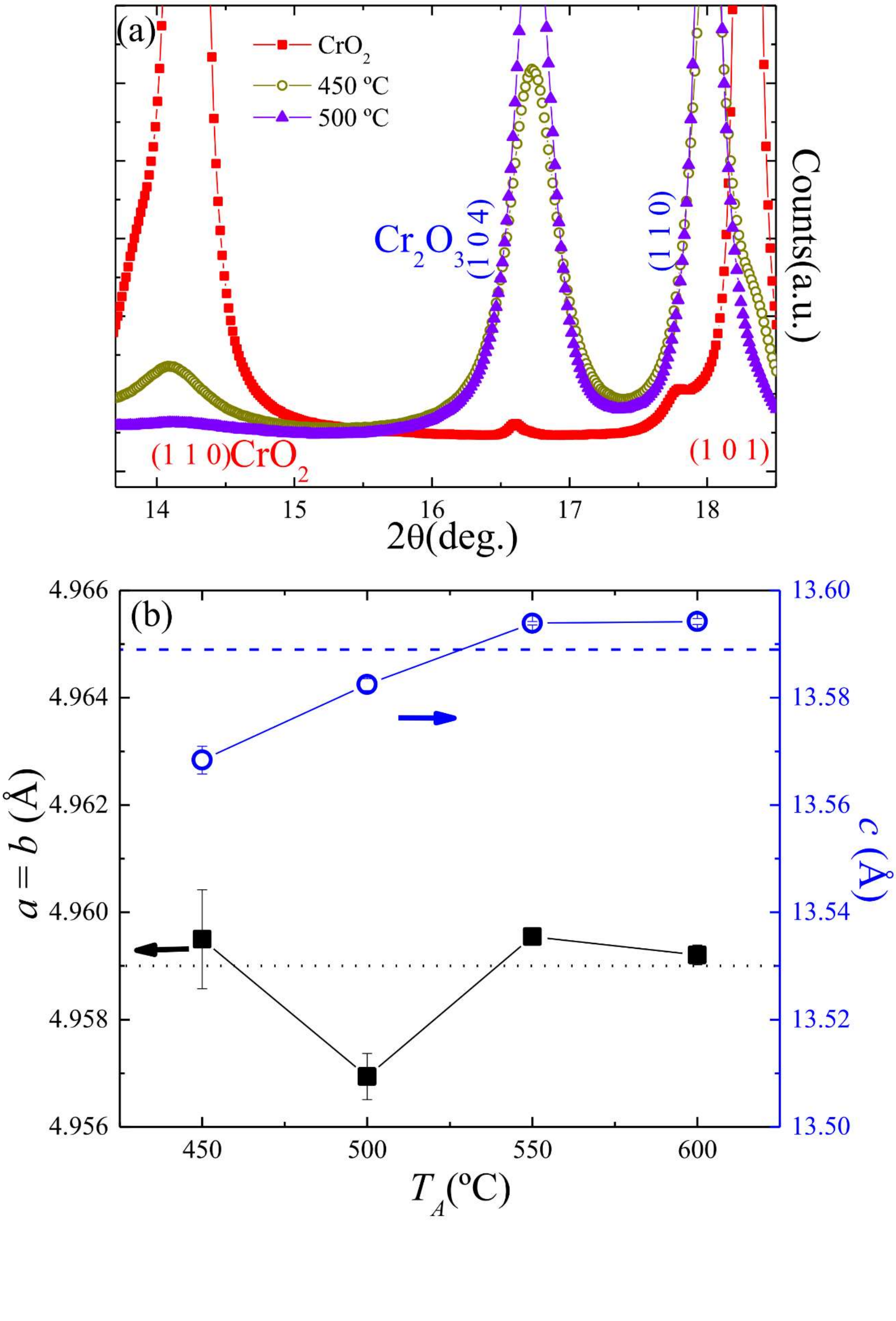

Table S1. Summary of the fitting parameters obtained from the Rietveld refined SRXRD spectra. All structural and lattice parameters were allowed to vary simultaneously, and the unweighted Rp, the weighted wRp factor, differed by less than one part in a thousand in two successive cycles.

Sample	Lattice constant (Å)				Wt. fraction (%)		wRp	Rp	χ^2
	CrO_2		Cr_2O_3		CrO_2	Cr_2O_3			
	a= *b*	*c*	*a* = *b*	*c*					
CrO_2	4.4215±0.0002	2.9177±0.0001			100	0	0.0861	0.0572	4.750
450 °C	4.4715±0.0027	2.9206±0.0023	4.9595± 0.0009	13.5684± 0.0026	0.327	99.673	0.1039	0.0847	5.270
500 °C			4.9537± 0.0005	13.5744± 0.0012	0	100	0.0790	0.0597	2.988
550 °C			4.9596± 0.0001	13.5939± 0.0003	0	100	0.0641	0.0451	1.802
600 °C			4.9592± 0.0002	13.5942± 0.0006	0	100	0.0603	0.0443	2.292
Cr_2O_3 NRs[28]			4.9667	13.6172	0	100	0.0548	0.0392	1.083

Table S2. Summary of saturation magnetization M_S measured at 2 K, mean diameter <*d*> of NRs, shell-Cr_2O_3 thickness t, core-CrO_2 diameter d_{core}, Curie temperature T_C, and fitting parameter α, respectively.

Sample	Ms(2K) (emu/g)	<*d*> (nm)	$t_{Cr_2O_3}$ (nm)	d_{Core}(nm)	T_C (K)	α
CrO_2	138.02	24±1	0	24	388±1	……
450	18.88	28±1	8.67	10.65	321±5	1.22±0.04
500	5.52	31±1	12.30	6.40	281±5	1.40±0.04
550	4.48	33±2	13.43	6.14	271±15	2.71±0.2
600	2.06	35±2	15.29	4.42	191±4	1.37±0.05
